# The Combined Use of Ozone and Negative Pressure Wound Therapy in the Management of Diabetes-Related Foot Disease: A Retrospective Exploratory Cohort Study

**DOI:** 10.3390/medicina62050827

**Published:** 2026-04-27

**Authors:** Izabella Kuźmiuk-Glembin, Agnieszka Białomyzy, Michał Sadowski, Bogdan Biedunkiewicz, Leszek Tylicki, Tomasz Niewęgłowski

**Affiliations:** 1 Department of Nephrology, Transplantology and Internal Diseases, Medical University of Gdansk, University Clinical Centre, 80-214 Gdansk, Poland; 2 ETER-MED, Wound Care Clinic, Non-Public Healthcare Centre, 80-822 Gdansk, Polandtn@etermed.pl (T.N.)

**Keywords:** ozone treatment, diabetic foot syndrome, negative pressure therapy, diabetes mellitus

## Abstract

*Background and Objectives*: Diabetes mellitus (DM) is a major global health concern, with diabetes-related foot disease (DFD) representing one of its most severe complications, often resulting in chronic infection, osteomyelitis, and limb amputation. Conventional therapies frequently fail in refractory cases, necessitating novel adjunctive strategies. Ozone therapy (OT) possesses antimicrobial, immunomodulatory, and oxygen-enhancing properties, while negative pressure wound therapy (NPWT) facilitates granulation, exudate removal, and tissue perfusion. This study explored the combined efficacy of OT and NPWT in advanced DFD. *Materials and Methods*: An exploratory, retrospective, observational cohort study was conducted at a specialized wound care center in Gdańsk, Poland, between 2019 and 2022. The study included 30 patients (*n* = 30) with refractory DFD involving both soft tissue and bone infection who had not responded to previous conventional treatment. The analyzed treatment approach consisted of surgical debridement, application of topical ozonated preparations, and (NPWT) with instillation of ozonated saline administered over a six-week period. Clinical outcomes included wound healing assessed using the Wagner classification and wound volume reduction, pain intensity measured using the Numeric Rating Scale (NRS), inflammatory biomarkers (C-reactive protein [CRP] and procalcitonin [PCT]), and microbiological characteristics of wound cultures. Statistical analyses were performed using the Wilcoxon signed-rank test and the chi-square test, and regression modeling was applied to identify potential predictors of therapeutic response. Statistical significance was defined as *p* < 0.05. *Results*: By week six, 100% of ulcers improved to Wagner stage ≤1, with 26.7% achieving stage 0. Median wound volume decreased from 5.5 cm^3^ to 0 cm^3^ (*p* < 0.001). Pain scores declined from 7.2 ± 0.96 points to 0.2 ± 0.5 points (*p* < 0.001). CRP and PCT levels decreased significantly (*p* < 0.001), and microbiological clearance was observed in all cases. Higher body mass index (BMI) was associated with poorer pain reduction. *Conclusions*: The combination of standard wound care with OT and NPWT was associated with clinically relevant improvements in wound healing, infection control, systemic inflammation, and pain reduction in patients with refractory DFD. Although limited by a non-controlled design and small cohort size, these findings support further randomized controlled trials to define the role of this combined approach in integrated diabetic foot care.

## 1. Introduction

Diabetes mellitus (DM) constitutes one of the most prevalent metabolic disorders, leading to hyperglycemia caused by either an absolute or relative deficiency of insulin. This condition precipitates a spectrum of severe complications, including macro- and microvascular damage, e.g., cardiovascular disease, retinopathy, neuropathy, nephropathy, as well as bone, joint, and skin complications, including diabetic foot disease [1]. The statistics are alarming—the International Diabetes Federation projects that the number of individuals living with diabetes will increase from 588.7 million in 2024 to over 852 million by 2050 [2]. The increasing prevalence of diabetes places a considerable strain on healthcare systems and national economies. Therefore, coordinated efforts in prevention, education, and treatment are necessary to mitigate its impact on individuals and society [3].

Diabetes-related foot disease (DFD) represents one of the most severe chronic complications of diabetes mellitus. According to the 2024 guidelines of the International Working Group on the Diabetic Foot (IWGDF), DFD is defined as a condition affecting the foot of an individual with current or previously diagnosed diabetes mellitus, characterized by one or more of the following: peripheral neuropathy, peripheral arterial disease, infection, ulceration, neuro-osteoarthropathy, gangrene, or amputation [4]. Reports indicate that the annual global incidence of DFD ranges from 1% to 4%, while the lifetime risk of developing a diabetic foot ulcer is estimated to be between 19% and 34% [5,6].

In Poland, DFD is also highly prevalent, with over 7% of individuals with diabetes exhibiting peripheral neuropathy, 24% presenting with foot deformities, and 17% showing signs of peripheral arterial disease [7]. Furthermore, the incidence of lower-extremity amputations—one of the most severe consequences of DFD—has increased significantly in parallel with the rising prevalence of diabetes in the population [8]. A recent meta-analysis reported an overall amputation rate of approximately 31% among patients with DFD. Several factors have been identified as significant predictors of lower-extremity amputation in this population, including male sex, smoking history, elevated body mass index (BMI), hypertension, cardiovascular disease, renal impairment, increased white blood cell count, and decreased haemoglobin and serum albumin levels [9]. Limb amputation should be regarded as a measure of last resort due to its substantial associated mortality, with reported 30-day postoperative mortality rates ranging from 7% to 51%, and a mean rate of approximately 16.5% [10].

Effective management of DFD requires accurate etiological differentiation and strict adherence to structured treatment strategies to prevent irreversible tissue damage and limb amputation. The TIME framework, developed by the European Wound Management Association, provides a comprehensive, evidence-based approach to wound management, integrating tissue debridement, infection and inflammation control, moisture balance, and wound edge advancement. This framework is complemented by conservative, surgical, and adjunctive therapies, including negative pressure wound therapy (NPWT), ozone therapy (OT), and hyperbaric oxygen therapy [11,12].

Ozone (O_3_) is a potent allotropic form of oxygen that has demonstrated significant antimicrobial, antiviral, and immunomodulatory properties, with a documented history of medical use spanning more than a century. NPWT complements OT by promoting granulation tissue formation, reducing oedema, and enhancing local perfusion. The synergistic application of OT and NPWT is hypothesized to enhance bacterial clearance and tissue regeneration and may therefore reduce healthcare costs, length of hospitalization, and reliance on systemic antibiotics and analgesics. Consequently, this multimodal approach may represent a promising therapeutic strategy for the management of chronic wounds and osteomyelitis associated with DFD, with the potential to significantly improve patient outcomes and quality of life.

This study aimed to conduct an exploratory clinical evaluation of the combined use of OT and NPWT in the treatment of DFD, with particular focus on wound healing, quantified by changes in wound volume. Secondary objectives included evaluating the effects of this therapeutic approach on the pain intensity assessed using the Numeric Rating Scale (NRS), inflammatory biomarkers, including C-reactive protein (CRP) and procalcitonin (PCT), as well as on the bacteriological status of the wound. The novelty of the present study does not lie in the isolated use of OT and NPWT as individual interventions, but rather in the exploratory real-world evaluation of their combined application within a structured multimodal treatment protocol implemented in a specialized wound care setting for patients with refractory DFD involving both soft tissue and bone infection. Given the retrospective and non-controlled design, the study was intended to generate preliminary clinical evidence regarding feasibility, safety, and potential signals of benefits.

## 2. Materials and Methods

This retrospective exploratory observational cohort study was conducted between 2019 and 2022 at the ETER-MED Centre for Chronic Wound Treatment in Gdańsk, Poland. Ethical approval was obtained from the Bioethics Committee of the Pomeranian Medical University (approval no. KB.006.130.2023, date: 20 December 2023). All participants provided written informed consent, and the study was conducted in accordance with the Declaration of Helsinki. In addition, written informed consent for publication of clinical photographs was obtained from patients whose images are included in the manuscript.

Patients included in the retrospective analysis were diagnosed with refractory DFD involving both soft tissue and bone infections localized to the toes and forefoot. For the purpose of this retrospective analysis, refractory DFD was defined as the absence of satisfactory clinical improvement after prior conventional wound management documented before referral to the study center. In most patients, such treatment had been continued for at least one month (70%), and in a substantial proportion for one year or longer (30%). The present cohort reflects the available real-world population treated with the ozone-based protocol during the study period and should not be interpreted as a entirely representative epidemiological sample of the broader DFD population. The treatment modalities applied previously are summarized in Table 1.

Subsequently, patients were managed according to the routine clinical practice of the center, which incorporated ozone-based therapy as part of the treatment strategy. The patients included in the present study constituted the first cohort at the center treated with these ozone-based interventions.

Patients with documented critical limb ischemia—defined at screening by an ankle–brachial index < 0.9 together with transcutaneous oxygen pressure (TcPO_2_) < 20 mmHg and Doppler ultrasound assessment—were excluded from the study. These vascular parameters were used primarily to determine eligibility and safety of local treatment; serial vascular measurements were not predefined study endpoints in this retrospective analysis.

### 2.1. Ozone and Negative Pressure Therapy Protocol

Following referral to our center, patients underwent multimodal wound management that included surgical debridement, standard wound care, followed by collection of wound specimens for microbiological culture and sensitivity testing. As well as systemic antibiotic therapy when clinically indicated. Repeated surgical resection of devitalized tissue was performed throughout the treatment period according to clinical need. After initial wound bed preparation, topical ozone-based therapy was commenced.

Ozonated olive oil, produced by cold-pressed olive oil saturation with ozone at a concentration of 70 µg/mL through repeated ozonation cycles, was applied directly to the wound surface in 2–3 application cycles using viscose-based dressings. In parallel, patients received optimized standard wound care, including antiseptic management and targeted systemic antibiotic therapy guided by microbiological findings. Repeated surgical resection of devitalized tissue was performed as clinically indicated throughout the treatment period.

Subsequently, NPWT with concurrent instillation and drainage of ozonated saline was initiated using a closed negative-pressure system (GENADYNA system) equipped with a multifunctional access port (Banasiewicz port). This system enabled simultaneous application of subatmospheric pressure and delivery of ozonated 0.9% sodium chloride solution, while maintaining a sealed wound environment. Ozonated saline was prepared by saturating 500 mL of 0.9% sodium chloride solution with an oxygen–ozone mixture at a target ozone concentration of 70 µg/mL for 30 min using a medical-grade ozone generator (ATO-3). The NPWT instillation protocol consisted of three sequential phases:

(1) instillation of ozonated saline via the port drain to fully saturate the polyurethane foam surrounding the wound;
(2) retention of the instilled fluid within the wound bed under controlled negative pressure for 5–10 min; and
(3) active removal of the irrigation fluid through suction.

Each treatment cycle lasted approximately 10–15 min. Particular attention was paid to maintaining the bactericidal activity window of ozone, as ozone molecules in 0.9% NaCl retain antimicrobial efficacy for approximately 15 min.

Negative pressure levels were maintained within a controlled range of 100–125 mmHg. Lower pressure values were avoided, as they do not improve therapeutic efficacy and may adversely affect local tissue perfusion, potentially leading to ischemic changes. NPWT with ozonated saline instillation was performed once daily for 10 consecutive days. After completion of this phase, standard NPWT without instillation was continued as clinically indicated.

Between NPWT sessions, all wounds were protected using specialized dressings impregnated with ozonated olive oil at a concentration of 70 µg/mL. The complete therapeutic protocol, including surgical, topical ozone, and NPWT-based interventions, was carried out over a total period of six weeks.

### 2.2. Wound Bed, Pain Intensity and Inflammation Assessment

Assessment of DFD was based on comprehensive review of patients’ medical records and clinical documentation maintained at the treatment centre. Clinical outcomes were evaluated at baseline and subsequently after 3 and 6 weeks of therapy. Wound status was assessed using the Wagner classification system and quantitative estimation of wound volume. Wound volume was calculated during routine clinical assessment using standardized manual measurements of maximal length, width, and depth (length × width × depth, expressed in cm^3^). To minimize inter-observer variability, all measurements were performed using the same standardized clinical approach by the same investigator throughout the study period. Wound exudate was recorded as a structured clinical characteristic based on routine wound assessment (e.g., purulent, dense, serous, scant, or absent) rather than by means of a validated quantitative exudate scale; therefore, exudate-related results are presented descriptively.

Pain intensity was assessed using the NRS scale, a validated 11-point numerical scale ranging from 0 (no pain) to 10 (worst imaginable pain). Furthermore, a structured subjective questionnaire was administered at baseline and at weeks 3 and 6 to characterize wound-related pain features, including acute, pulsatile, chronic, mild, moderate, and sharp pain.

Systemic inflammation and infection were evaluated using laboratory biomarkers, including serum CRP and PCT, alongside microbiological analysis of wound swab specimens. Glycaemic control was assessed by measurement of glycated haemoglobin (HbA1c). Microbiological assessment was based on wound swab specimens obtained as part of routine clinical care. Antibiotic therapy was administered when clinically indicated, based on routine clinical decision-making and guided by microbiological findings. The selection of antimicrobial agents was clinician-dependent rather than protocol-standardized and reflected real-world practice. Consequently, antibiotic exposure varied between patients and formed part of the overall multimodal treatment strategy.

### 2.3. Statistical Analysis

Statistical analyses were performed using Stata/BE version 18 (StataCorp LLC, College Station, TX, USA). Given the retrospective exploratory design and the limited sample size, no prospective sample size calculation was performed, and all analyses were intended to provide descriptive and hypothesis-generating insights rather than confirmatory evidence.

Continuous variables were summarized as mean ± standard deviation (SD) or median with interquartile range (IQR), as appropriate based on data distribution. Categorical variables were reported as absolute frequencies and percentages. Normality of continuous variables was assessed using the Shapiro–Wilk test in conjunction with graphical distribution diagnostics. Variables that did not meet normality assumptions were analysed using nonparametric statistical methods.

Repeated measurements obtained at baseline, week 3, and week 6 were analysed using pairwise comparisons with the Wilcoxon signed-rank test. This approach was selected due to the small sample size and non-normal distribution of several variables, and to assess within-patient changes over time in an exploratory manner. Comparisons of ordinal and categorical variables were performed using the chi-square (χ^2^) test, as appropriate. No formal correction for multiple comparisons was applied; therefore, *p* values should be interpreted with caution and considered descriptive.

To explore potential predictors of clinical response, linear regression models were constructed to assess associations between selected patient- and disease-related variables and clinical outcomes. Given the limited sample size and the potential for collinearity among variables, these analyses were considered exploratory and underpowered, and their results should be interpreted with caution. Regression results are presented as regression coefficients (β) with corresponding *p* values, and confidence intervals are reported for regression analyses. No correction for multiple comparisons was applied; therefore, *p* values should be interpreted as descriptive.

All statistical tests were two-tailed, and statistical significance was defined as *p* < 0.05.

## 3. Results

Within the study cohort, 80% of participants were male (24 men, 6 women), and the mean age was 66.9 ± 12.3 years. The majority of patients (80%, n = 24) were diagnosed with type 1 diabetes mellitus, while the remaining 20% (n = 6) had type 2 diabetes mellitus. The predominance of patients with type 1 diabetes in this cohort reflects the referral pattern of the specialized center and should be interpreted with caution when considering external validity, as most published diabetic foot cohorts are dominated by patients with type 2 diabetes. Insulin therapy was required in 80% of cases, whereas 20% of participants were insulin-independent. At baseline, glycaemic control was suboptimal, with a median glycated haemoglobin (HbA1c) level of 7.15% (interquartile range [IQR]: 6.7–8.2).

The most prevalent comorbidities were diabetes-related complications, predominantly cardiovascular disease, arterial hypertension, anaemia, gastrointestinal disorders, and neurological impairments. All participants exhibited reduced functional mobility and required assistive devices: 10% (n = 3) used a walker, 83.3% (n = 25) used crutches, 16.6% (n = 5) were wheelchair-dependent, 60% (n = 18) used a forefoot offloading shoe, and 6.7% (n = 2) used a hindfoot offloading shoe. Detailed demographic and clinical characteristics of the study population are summarized in Table 2.

### 3.1. Wound Healing Outcomes

Assessment of ulcer severity using the Wagner classification system demonstrated progressive and statistically significant improvement over the course of treatment. At baseline, the majority of patients presented with advanced ulcerations classified as Wagner stages 3–5. By week 6, 73.3% of patients were classified as Wagner stage 1, and 26.7% as stage 0, with no patients remaining in stages 3–5. These changes were statistically significant across multiple time-point comparisons (*p* < 0.05). Detailed assessments of wound characteristics and staging are presented in Table 3 and Table 4.

**Figure 1:**
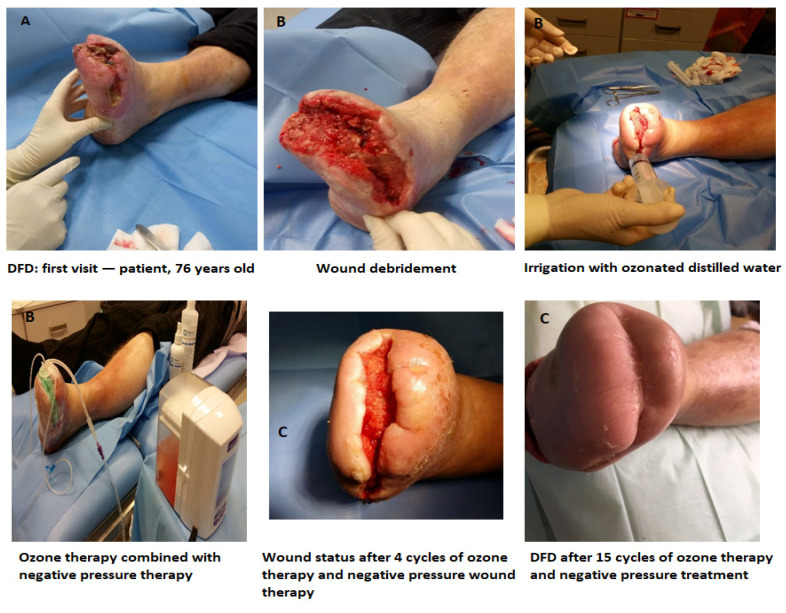
Representative images of diabetic foot ulcers before treatment (**A**), during therapy (**B**), and after completion of treatment (**C**).

Wound volume decreased significantly over the course of treatment, from a baseline median of 5.5 cm^3^ (IQR: 2.0–23.97) to 0.4 cm^3^ (IQR: 0.15–1.5) at week 3 (*p* < 0.001), and further to 0 cm^3^ at week 6 (*p* < 0.001). In more than 93% of patients, the wound bed demonstrated a structured progression from the inflammatory and destructive phase through the proliferative and reparative phase to the remodeling and consolidation phase of wound healing. Exudate characteristics evolved in parallel, transitioning from predominantly purulent and dense secretions at baseline to mainly clear, serous, or absent drainage by week 6 (χ^2^ test, *p* < 0.05). These findings are illustrated in Figure 1.

No major adverse events of OT and NPWT were noted.

### 3.2. Pain Intensity Outcomes

**Figure 2:**
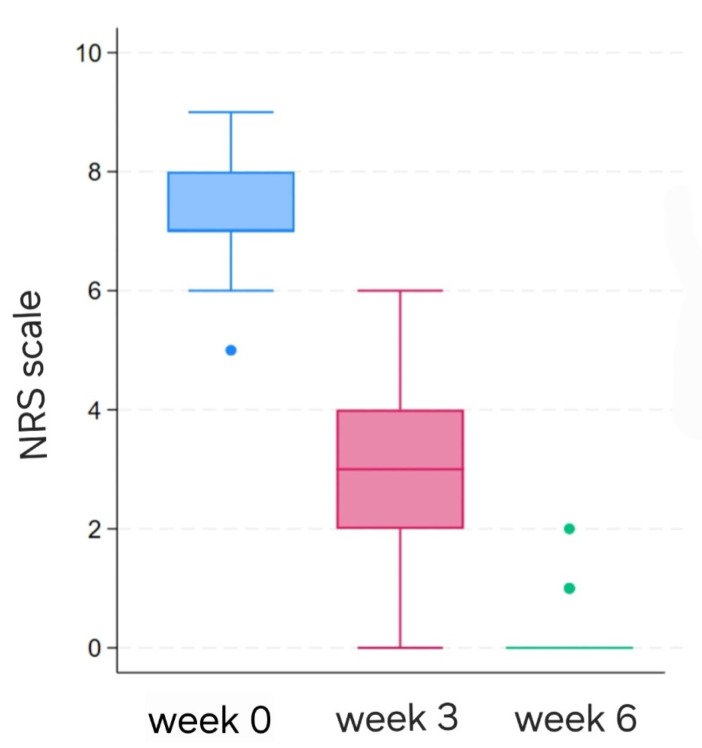
The pain intensity changes in NRS scale.

Pain intensity, assessed using the NRS scale and analyzed as a secondary patient-reported outcome, demonstrated a marked reduction over the study period. Statistically significant improvements were observed between baseline and week 3 (mean ± SD: 7.2 ± 0.96 vs. 3.0 ± 1.1; *p* < 0.001), as well as between week 3 and week 6 (3.0 ± 1.1 vs. 0.2 ± 0.5; *p* < 0.001) (Figure 2). Pain characteristics evolved from predominantly sharp and pulsatile at baseline to intermittent and mild by week 6, with only one patient reporting occasional discomfort.

However, given the high prevalence of diabetic neuropathy in the cohort, pain should not be interpreted as a standalone objective marker of wound improvement.

### 3.3. Inflammatory Parameters and Wound Infection

**Figure 3:**
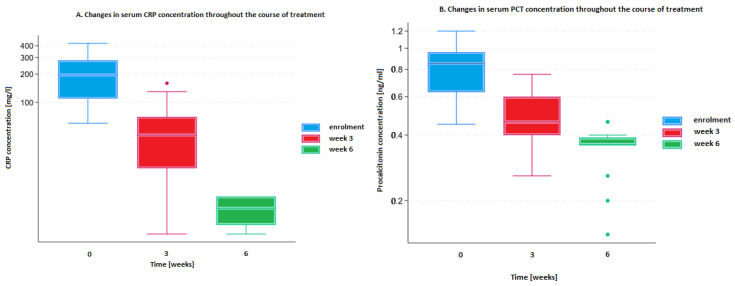
Effects of treatment on serum CRP (**A**) and PCT (**B**) concentrations over the study period.

Longitudinal analyses demonstrated a pronounced and statistically significant decline in inflammatory biomarkers over the treatment period. Median CRP levels decreased from 195.5 mg/L (IQR: 110–280) at baseline to 45 mg/L (IQR: 20–70) at week 3, and further to 7.5 mg/L (IQR: 5–10) at week 6 (*p* < 0.001 for both comparisons). Similarly, median PCT concentrations declined from 0.75 ng/mL (IQR: 0.63–0.96) at baseline to 0.46 ng/mL (IQR: 0.40–0.60) at week 3, and to 0.36 ng/mL (IQR: 0.36–0.39) at week 6 (*p* < 0.001 for both comparisons). Both CRP and PCT exhibited consistent downward trends throughout the treatment period, in line with resolution of systemic and local inflammatory responses (Figure 3A,B). These changes should be interpreted in the context of the multimodal treatment approach, including surgical debridement and systemic antibiotic therapy in a substantial proportion of patients.

At baseline, wound infections were present in all patients. The majority of participants (>80%) exhibited clinical signs of local infection, including pain, oedema, periwound erythema, malodorous discharge, local fistula formation, purulent exudate, and osteomyelitis. The presence of soft tissue and bone infection, including suspected osteomyelitis, was determined based on clinical assessment and available medical documentation at the time of treatment. Given the retrospective design of the study, a uniform diagnostic algorithm (e.g., mandatory radiological, microbiological, or surgical confirmation) was not applied across all patients, and diagnoses reflect routine clinical practice rather than standardized study criteria. Approximately 30% of patients also reported systemic or advanced features, such as impaired wound healing, fever, generalized weakness, and the presence of bone fragments within the wound.

Microbiological analysis of wound swabs obtained at enrolment revealed predominant colonization by *Pseudomonas aeruginosa* (n = 28; 93.3%), *Staphylococcus aureus* (n = 8; 26.7%), and *Enterococcus faecalis* (n = 4; 13.3%). Less frequently isolated pathogens included *Staphylococcus agalactiae*, *Proteus mirabilis*, and *Escherichia coli*, each identified in a single patient (3.3%).

In addition to OT and NPWT, systemic antibiotic therapy was administered to 18 patients (60%), guided by microbiological findings. The most commonly prescribed agents were amoxicillin (n = 6; 23.3%), clindamycin and trimethoprim/sulfamethoxazole (each n = 3; 10%), ciprofloxacin or levofloxacin (each n = 2; 6.7%), and gentamicin or cefuroxime (each n = 1; 3.3%).

After six weeks of treatment, no patients exhibited clinical signs of either local or systemic infection. Granulation tissue formation and progression toward wound healing were observed in 28 patients (93.3%). Follow-up microbiological cultures confirmed eradication of pathogenic organisms in all cases with only physiological flora detected in all patients (*p* < 0.05).

### 3.4. Regression Analyses

In regression models evaluating predictors of wound healing, none of the baseline laboratory parameters, including CRP, PCT, or glycated haemoglobin, nor clinical variables such as age, smoking status, cardiovascular comorbidities, or baseline wound volume, demonstrated statistically significant associations with wound volume reduction. Furthermore, individual therapeutic components, including OT and NPWT–related procedures, exhibited substantial collinearity, which precluded reliable estimation of their independent effects within multivariable models. Consequently, regression analyses of wound healing outcomes were not considered statistically robust and were interpreted descriptively.

In exploratory regression analysis, BMI was found to be associated with changes in pain scores. However, given the limited sample size and potential model instability, this finding should be interpreted with caution and considered hypothesis-generating. BMI demonstrated a positive linear association with pain outcomes, indicating that higher BMI was associated with less favourable pain reduction (β = 0.25 per BMI unit; 95% confidence interval [CI]: 0.13–0.37; *p* = 0.003, adjusted R^2^ = 0.750). Other examined variables, including baseline inflammatory markers (CRP), baseline wound volume, and glycaemic control, were not significantly associated with pain reduction. Although baseline CRP exhibited a non-significant trend toward association with pain outcomes (*p* = 0.132), this did not reach statistical significance.

Given the exploratory design, limited sample size, and the presence of collinearity between therapeutic interventions, regression analyses were intended to generate hypotheses rather than support definitive causal inference. These findings underscore the need for future controlled studies with parallel treatment arms and adequate statistical power to disentangle the independent and synergistic effects of OT and NPWT.

## 4. Discussion

Within the TIME framework, effective management of DFD requires systematic removal of necrotic tissue—whether surgical, enzymatic, biological, or vacuum-assisted—to restore wound bed viability. Infection control relies on microbiologically guided antimicrobial therapy, antiseptics, and advanced wound dressings, with adjunctive options such as maggot therapy or vacuum-assisted techniques reserved for severe or refractory cases. Maintenance of optimal moisture balance is equally critical, as both desiccation and excessive exudate impair cellular migration and tissue repair.

Non-healing ulcers necessitate reassessment of the initial diagnosis, exclusion of systemic comorbidities or malignancy, and consideration of adjunctive therapeutic strategies. Conservative management focuses on effective pressure offloading and optimization of tissue perfusion, often requiring revascularization procedures and prolonged antimicrobial treatment. In cases of osteomyelitis, surgical debridement of infected bone and soft tissue remains a cornerstone of therapy, supported by local antimicrobial carriers and biomaterials with osteoconductive and osteoinductive properties [11]. In accordance with contemporary clinical practice, failure of standard wound care is typically determined after a minimum of six weeks of optimized therapy.

Despite multidisciplinary management, global healing outcomes for diabetic foot ulcers remain suboptimal. A recent meta-analysis of randomized controlled trials reported a pooled ulcer closure rate of 33.1% at 12–24 weeks, while real-world data indicate that fewer than half of patients achieve complete healing within 12 weeks [13]. Moreover, treatment durability is limited, with pooled recurrence rates estimated at 22.1% per person-year (95%CI: 19.0–25.2%), and recurrence rates reported to reach up to 70% within 10 years following initial healing [14]. Importantly, the clinical consequences of non-healing ulcers are substantial: a 2024 meta-analysis estimated an overall lower-extremity amputation rate of approximately one third among patients with diabetic foot ulcers, underscoring the need for timely recognition of standard-care failure and early transition to adjunctive or advanced therapeutic approaches.

NPWT and OT, as advanced therapeutic modalities, represent promising adjuncts to standard care by enhancing tissue repair and reducing the risk of infection recurrence. These approaches constitute important and emerging directions in the management of DFD, particularly in the context of the rapidly increasing global burden of diabetes mellitus and the substantial proportion of patients who fail to respond to conventional treatment strategies.

The clinical application of ozone in the treatment of chronic wounds, including DFD, is supported by its multifaceted biological effects, including disruption of microbial cell membranes, modulation of innate and adaptive immune responses, and enhancement of tissue oxygenation through complex biochemical pathways. Ozone has been shown to stimulate erythrocyte glycolysis, increase intracellular levels of 2,3-diphosphoglycerate, and thereby improve oxygen release to ischemic tissues. In addition, ozone promotes vasodilation and enhances adenosine triphosphate (ATP) production via activation of the Krebs cycle [15]. Clinical studies have demonstrated that OT can accelerate wound healing by enhancing granulation, tissue formation, reducing oxidative stress, and increasing the expression of growth factors such as platelet-derived growth factor (PDGF) and transforming growth factor β1 (TGF-β1) [16,17,18].

In the context of bone and joint infections that are refractory to conventional therapies, ozone has been shown to enhance local immune responses and reduce bacterial resistance, supporting its role as a valuable adjunct in orthopaedic and surgical infection management. NPWT complements the biological effects of OT by generating controlled subatmospheric pressure within the wound bed, thereby promoting granulation and tissue formation through upregulation of the hypoxia-inducible factor 1α (HIF-1α)/vascular endothelial growth factor (VEGF) pathway, reducing interstitial oedema, and improving capillary density and tissue oxygenation [19]. The combined use of NPWT with ozone irrigation, such as instillation of ozonated saline, has been reported to facilitate wound sterilization and epithelialization, particularly in complex or non-healing ulcers [20].

Clinical protocols emphasize the importance of individualized ozone dosing, typically ranging from 20 to 75 μg/mL, and tailoring NPWT parameters—most commonly subatmospheric pressures between 80 and 125 mmHg—to optimize therapeutic efficacy while ensuring patient safety.

The present exploratory study suggests that the combined application of OT and NPWT was associated with clinically relevant improvement in patients with refractory DFD treated in a specialized center. The observed changes included improvement in wound stage, reduction in wound volume, decline in inflammatory biomarkers, and improvement in microbiological findings and pain scores. However, given the retrospective non-controlled design and the multimodal nature of treatment, these associations should be interpreted cautiously and regarded as hypothesis-generating rather than confirmatory.

Collectively, these findings add to the growing body of evidence suggesting that multimodal therapeutic strategies integrating advanced wound care technologies may substantially improve clinical outcomes in patients with chronic, treatment-refractory diabetic foot ulcers.

Wound healing followed a favourable and structured trajectory in the majority of patients included in this study. By week 6, nearly all ulcers had transitioned from advanced Wagner stages (3–5) to superficial lesions or complete epithelialization. This progression closely mirrors the physiological phases of wound healing, with a transition from inflammation through proliferation to remodelling, underscoring the potential of this therapeutic strategy to restore endogenous repair mechanisms even in previously non-healing ulcers. The marked reduction in wound volume—from a median of 5.5 cm^3^ at baseline to complete closure in most cases—is potentially, clinically meaningful and exceeds outcomes typically reported with conventional dressings or systemic antibiotic therapy alone, which are frequently limited by impaired perfusion, antimicrobial resistance, and biofilm formation. However, these findings cannot be attributed only and specifically to OT and NPWT alone, as systemic antibiotic therapy and repeated debridement were also part of the treatment strategy.

These findings are consistent with existing evidence supporting the efficacy of NPWT in diabetic foot ulcers. A systematic review of 11 randomized controlled trials involving 1044 patients demonstrated that NPWT significantly improved healing outcomes compared with standard dressings, resulting in higher closure rates, faster wound resolution, greater reductions in ulcer size and depth, and fewer amputations, without an increase in adverse events [21]. Similarly, a meta-analysis encompassing 17 studies evaluating ozone therapy (OT) reported comparable reductions in wound size and favourable effects on wound microbiological status [22]. The concurrent improvements observed in exudate characteristics, microbiological clearance, and wound volume in the present cohort support a synergistic interaction between OT and NPWT, whereby NPWT promotes angiogenesis and granulation tissue formation, while ozone provides antimicrobial and immunomodulatory effects. The convergence of these mechanisms likely accounts for the rapid wound stabilization observed.

In the study a reduction in reported pain intensity during the treatment period was observed; however, this finding should be interpreted with caution, as patients with DFD frequently present with peripheral neuropathy, which may alter pain perception and limit the reliability of pain as an objective indicator of clinical improvement. Nevertheless, pain is frequently underreported as a clinical endpoint in studies of DFD, despite its substantial impact on mobility, quality of life, and treatment adherence. The transition from severe baseline pain (mean NRS > 7) to minimal or absent pain by week 6 is particularly noteworthy, given that pain associated with DFD often has a neuropathic and ischemic component and is frequently refractory to standard analgesic strategies. Regression analysis identified higher body mass index (BMI) as a potential factor associated with less pronounced pain reduction, in line with existing literature linking obesity to chronic low-grade inflammation, mechanical overload, and impaired tissue repair. Comparable studies employing the visual analogue scale (VAS) have similarly demonstrated significant pain reductions following OT, although variability in effect size and methodological limitations, including small sample sizes and single-centre designs, underscore the need for further validation [23,24]. These observations highlight the multifactorial nature of pain in DFD (including wound healing, reduced inflammation, optimized offloading, and non-specific effects) and emphasize the importance of addressing systemic metabolic factors alongside local wound-directed therapies. Noteworthy is the role of analgesic management in pain outcomes, which should be systematically documented in future studies and follow-up protocols to allow accurate interpretation of treatment-related pain reduction.

Equally important was the substantial improvement in inflammatory biomarkers observed in this study. Both CRP and PCT demonstrated statistically and clinically significant reductions over the six-week treatment period, consistent with resolution of local and systemic inflammatory processes. These findings align with the established antimicrobial and immunomodulatory properties of ozone, which include disruption of bacterial cell membranes, enhancement of tissue oxygenation, and modulation of host immune responses. The complete eradication of pathogenic microorganisms—most notably *Pseudomonas aeruginosa* and *Staphylococcus aureus*, which are commonly implicated in recalcitrant diabetic foot infections—further supports the antibacterial efficacy of the combined therapeutic protocol. Similar results have been reported in smaller clinical studies, in which adjunctive OT was associated with reduced bacterial burden and shortened time to wound sterilization [25]. Although causal inference is precluded by the non-controlled design of the present study, the consistent and clinically meaningful improvements observed over a relatively short six-week period in a cohort refractory to prolonged conventional therapy are unlikely to be attributable solely to standard wound care and should therefore be regarded as hypothesis-generating, warranting confirmation in controlled trials.

From a clinical standpoint, the combined application of OT and NPWT offers several potential advantages in the management of DFD. First, effective control of bacterial load and systemic inflammation may reduce the need for prolonged systemic antibiotic therapy, thereby limiting the risks of antimicrobial resistance and treatment-related toxicity. Second, the observed improvements in pain and functional status may translate into enhanced patient mobility and quality of life—outcomes that are often insufficiently captured in clinical trials. Finally, the relative technical simplicity of OT, particularly when integrated with NPWT, may facilitate broader implementation of this approach in specialized wound care settings; however, its cost-effectiveness remains to be formally evaluated in future controlled studies incorporating health economic analyses.

The present findings should be interpreted within the context of real-world clinical practice and are intended to complement, rather than replace, evidence derived from controlled trials.

### Limitations and Future Directions

Several limitations of this study should be acknowledged. First, the exploratory, non-controlled design and relatively small sample size limit the generalizability of the findings and preclude causal inference. However, given the refractory nature of DFD in the studied population and the paucity of effective treatment options in this clinical context, an exploratory approach was considered appropriate to generate preliminary evidence regarding the feasibility, safety, and potential clinical benefit of combining OT with NPWT. Such hypothesis-generating studies represent an important step prior to the design of adequately powered randomized controlled trials. Second, the absence of a parallel control group prevents definitive attribution of the observed clinical improvements exclusively to the combined OT and NPWT intervention, as some degree of wound healing may occur with optimized standard care alone. Moreover, the cohort was clinically heterogeneous with respect to Wagner stage, ulcer duration, and comorbidity burden. Third, the treatment protocol was multimodal and included surgical debridement, standard wound care, and systemic antibiotic therapy in a substantial proportion of patients, which precludes disentangling the independent effects of OT and NPWT. In addition, the concurrent application of both therapies may have introduced collinearity between interventions, further limiting the ability to isolate their individual contributions to wound healing and infection control.

In observational settings, advanced statistical approaches may further strengthen causal inference. The use of propensity score matching could allow comparison between patients treated with OT plus NPWT and historical or contemporaneous cohorts receiving NPWT alone, while controlling for key confounding variables such as age, diabetes duration, ischemia severity, presence of neuropathy, wound size, and comorbidities. Additionally, mixed-effects models could account for repeated measurements over time and patient-level heterogeneity, while mediation analyses or structural equation modelling may help elucidate indirect pathways through which OT influences clinical outcomes, such as reductions in bacterial burden or modulation of inflammatory responses.

Another limitation is the lack of mechanistic endpoints. Incorporating biological markers as secondary outcomes in future studies could provide deeper insight into the molecular and cellular mechanisms underlying the observed clinical effects. Local assessments of oxidative stress markers, inflammatory cytokines, and angiogenic or proliferative growth factors such as vascular endothelial growth factor (VEGF) and platelet-derived growth factor (PDGF) may clarify how controlled ozone-induced oxidative stress influences the wound microenvironment.

Finally, the relatively short follow-up period limits assessment of long-term outcomes, including ulcer recurrence, durability of healing, limb salvage, and survival. Future protocols should incorporate extended follow-up of at least 6–12 months and include standardized patient-reported outcome measures, such as the SF-36 or Diabetic Foot Ulcer Scale, to capture quality-of-life benefits. Broader inclusion of patients with different types of diabetes, diverse ethnic backgrounds, and a wider spectrum of comorbid conditions would further enhance external validity and clinical applicability.

## 5. Conclusions

In summary, this retrospective exploratory study suggests that the combined use of OT and NPWT may be safe, feasible, and potentially effective in the management of DFD. The protocol was associated with marked reductions in infection, systemic inflammation, wound size, and pain intensity, with the majority of patients demonstrating progression to advanced stages of wound healing within six weeks. While these findings should be regarded as preliminary and hypothesis-generating as well as interpreted with caution due to methodological limitations, they underscore the potential of multimodal, biologically targeted therapies to address the complex pathophysiology of DFD. Further rigorously designed prospective trials are warranted to confirm these benefits and to determine the optimal role of OT and NPWT within integrated diabetic foot care pathways.

## Figures and Tables

**Table 1:** Treatments received prior to referral to the study center and before initiation of the study protocol.

Method	n (%)
**Disinfection:**	30 (100)
- octenidine	28 (93.3)
- polyhexanide	2 (6.7)
- povidone-iodine solution	2 (6.7)
- sodium hypochlorite	0 (0)
**Wound Debridement** (scalpel or surgical spoon)	6 (20)
**Ointments:**	19 (63.3)
- Betadine	11 (36.7)
- Iruxol	8 (26.7)
**Gels for Wound Debridement** **:**	15 (50)
- Granudacyn	5 (16.7)
- Microdacyn	1 (3.3)
- Intrasite	1 (3.3)
- Medisorb	2 (6.7)
**Dressings:**	30 (100)
Hydrocolloid Dressings:	15 (50)
- Granuflex	14 (46.7)
- Hydrocoll	1 (3.3)
Dressings Silver Ion	26 (86.7)
Manuka Honey Dressings	0 (0)
Iodine-Containing Inadine Dressing	2 (6.7)
Absorbent Dressings	30 (100)
- gauze and sterile compresses	29 (96.7)
- Zetuvit	4 (13.3)
- Mepilex	3 (10)
- Aquacel Foam	2 (6.7)
- Mepilex Border	1 (3.3)
- Suprasorb	3 (10)
- Sorbact	1 (3.3)
- Biatain	1 (3.3)
**Other:**	0 (0)
- biological dressings (larval therapy)	0 (0)
- negative pressure wound therapy	0 (0)
- ozone therapy	0 (0)
- hyperbaric chamber treatment	0 (0)

**Table 2:** General characteristics of the study cohort.

	Mean (± SD)/n (%)
**Age (years)**	66.9 (±12.3)
**Sex (M/F)**	24 (80)/6 (20)
**BMI (kg/m^2^)**	28.6 (±4.2)
**DM type 1/DM type 2**	24 (80)/6 (20)
**Cardiovascular:**	
- chronic coronary syndrome	17 (56.7)
- myocardial infarction in history	8 (26.7)
- chronic heart insufficiency	3 (10)
- arrhythmias	22 (73.3)
- arterial hypertension	25 (83.3)
- chronic venous insufficiency	11 (36.7)
**Haemathologic:**	
- anaemia	14 (46.7)
- leukopenia	0 (0)
- leukaemia	1 (3.3)
- lymphoma	1 (3.3)
**Pulmonary:**	
- asthma	6 (20)
**Hypothyroidism**	3 (10)
**Gastrointestinal:**	
- gastric ulcer disease	12 (40)
- duodenal ulcer disease	7 (23.3)
- cholelithiasis	6 (20)
- gastroesophageal reflux disease	15 (50)
- chronic diarrhoea	3 (10)
**Hepatic Cirhosis**	4 (13.3)
**Nephrotic Proteinuria**	4 (13.3)
**Urinary incontinence**	8 (26.7)
**Urinary tract neoplasms**	5 (16.7)
**Gout**	8 (26.7)
**Neurological:**	
- peripheral neuropathy	29 (96.7)
- polyneuropathy	23 (76.7)
- paraplegia	1 (3.3)
- tetraplegia	0 (0)
- depression	17 (56.7)

**Table 3:** Clinical evaluation of ulcerations before initiation of therapy.

Clinical Evaluation (A)	n (%)
**Duration of ulcer:**	
- One year or more	9 (30)
- One month or more	20 (66.7)
- One week or more	1 (3.3)
**Location of ulcer:**	
- On the plantar surface	14 (46.7)
- On the forefoot	8 (26.7)
- On the hallux (big toe)	18 (60)
- On toes II, III, IV, V	21 (70)
**Other characteristic features:**	
- No pain or mild pain	2 (6.7)
- Sensory disturbances	30 (100)
- Foot normally warm and pink	20 (66.7)
- Palpable pulse in the foot	30 (100)

**Table 4:** Assessment of ulceration according to the Wagner classification during the course of treatment.

Grade	Description	0n (%)	3 Weeks n (%)	6 Weeks n (%)	*p*
**0**	Structural deformity of the foot without overt ulceration; limb considered at high risk of developing diabetic foot syndrome	0 (0)	0 (0)	8 (26.7)	**<0.05**
**1**	Presence of a superficial ulcer confined to the epidermis and/or dermis	0 (0)	4 (13.3)	22 (73.3)	**<0.05**
**2**	Ulcer associated with localized infection involving the skin and subcutaneous tissue	0 (0)	24 (80)	0 (0)	**<0.001**
**3**	Deep ulcer extending to bone and/or joint, frequently complicated by phlegmon or deep tissue infection	18 (60)	1 (3.3)	0 (0)	**<0.001**
**4**	Localized gangrene of the toes or heel	7 (23.3)	1 (3.3)	0 (0)	**<0.05**
**5**	Extensive gangrene with systemic involvement (sepsis), typically requiring major amputation	5 (16.7)	0 (0)	0 (0)	**<0.05**

## Data Availability

The data presented in this study are available on request from the corresponding author.

## Abbreviations

The following abbreviations are used in this manuscript:

BMI	body mass index
CRP	C-reactive protein
DFD	diabetic foot disease
DM	diabetes mellitus
HbA1c	glycated hemoglobin
NPWT	negative pressure wound therapy
NRS	numeric rating scale
OT	ozone therapy
PCT	procalcitonin
TcPO_2_	transcutaneous oxygen pressure
